# Effectiveness of slightly acidic electrolyzed water on bacteria reduction: in vitro and spray evaluation

**DOI:** 10.7717/peerj.8593

**Published:** 2020-02-18

**Authors:** Angelica Naka, Masaya Yakubo, Kenji Nakamura, Midori Kurahashi

**Affiliations:** 1 Graduate School of Agricultural and Life Sciences, The University of Tokyo, Tokyo, Japan; 2 Research Department, Toyotomi Co. Ltd., Nagoya, Japan

**Keywords:** Slightly acidic electrolyzed water, Bacteria, Disinfection, Food preservation, In vitro, Spray

## Abstract

Bacterial inactivation is a crucial aspect of sanitation and hygiene. The effectiveness of slightly acidic electrolyzed water (SAEW) for reduction or removal of *Escherichia coli*, *Pseudomonas aeruginosa* and *Staphylococcus epidermidis* was evaluated. The bactericidal activity of SAEW and sodium hypochlorite (NaOCl) against *E. coli* and *P. aeruginosa* were compared through in vitro experiments. The effectiveness of SAEW spray was tested against *S. epidermidis*. Results showed that SAEW had a more powerful bactericidal activity than NaOCl at the same available chlorine concentrations. For *E. coli*, SAEW decreased the bacterial counts from 8.4 log_10_ CFU/mL to less than 3.9 log_10_ CFU/mL; NaOCl with the same available chlorine of 0.5 mg/L, caused a decrease from 8.4 log_10_ CFU/mL to 7.1 log_10_ CFU/mL. For *P. aeruginosa*, SAEW caused bacterial counts to decrease from 8.5 log_10_ CFU/mL to less than 4.1 log_10_ CFU/mL against 8.5 log_10_ CFU/mL to 6.2 log_10_ CFU/mL for NaOCl with the same available chlorine of 0.5 mg/L. Spray experiments showed that 10 mg/L of SAEW spray decreased the bacterial counts of *S. epidermidis* from 3.7 log_10_ CFU/m^3^ to 2.8 log_10_ CFU/m^3^, with 20 mg/L causing a reduction from 3.8 log_10_ CFU/m^3^ to 0 CFU/m^3^. The overall findings of this study indicate that SAEW may be a promising disinfectant agent either as a solution or spray.

## Introduction

Sanitization and hygiene play an important role in food preservation and consumption because some microorganisms such as bacteria can be harmful to the health of people, animals or plants. Bacteria are highly adaptable and have low nutrient requirements, making them available nearly everywhere with enough water. They are therefore well known as a source of contamination in tap water and raw or slightly cooked food. This contamination can cause dangerous infections in people with immunodeficiency or open wounds. Numerous sanitizers, such as sodium hypochlorite, chlorine dioxide, hydrogen peroxide, organic acids and ozone, have been extensively used for their effectiveness in reducing or removing pathogenic bacteria in agriculture and the food industry, markets, hospitals, care homes, layer housing environments, households, etc. (Koide et al., 2011). However, the majority of chemical disinfectants in use are difficult to handle, potentially toxic, corrosive, volatile and often not very effective (Forghani & Oh, 2013; Koseki et al., 2004).

Thus, the use of slightly acidic electrolyzed water (SAEW) has been introduced as an alternative and novel method to decontaminate surface and airborne pathogenic microorganisms. In Japan, SAEW has been authorized as: a food additive by the Japanese Ministry of Health, Labor and Welfare (June 2002); a specific pesticide by the Japanese Ministry of the Environment and the Ministry of Agriculture, Forestry and Fisheries (March 2014); and a cultivation material of organic JAS agricultural products by the Japanese Ministry of Agriculture, Forestry and Fisheries (March 2014). In Japan, the current maximum allowed concentration of free chlorine in SAEW is 80 mg/L. SAEW has been applied in Japan in food line production, hospitals, nursing homes, kindergartens, restaurants and other places where high levels of personal hygiene management are required. It is used as a substitute for sanitizers and deodorizers containing hazardous chemical materials and, thus, prevent accidental ingestion and handling of hazardous chemicals. With climate change and the subsequent risk of increasing airborne infectious diseases, there is a growing opportunity for the SAEW market.

**Figure 1:**
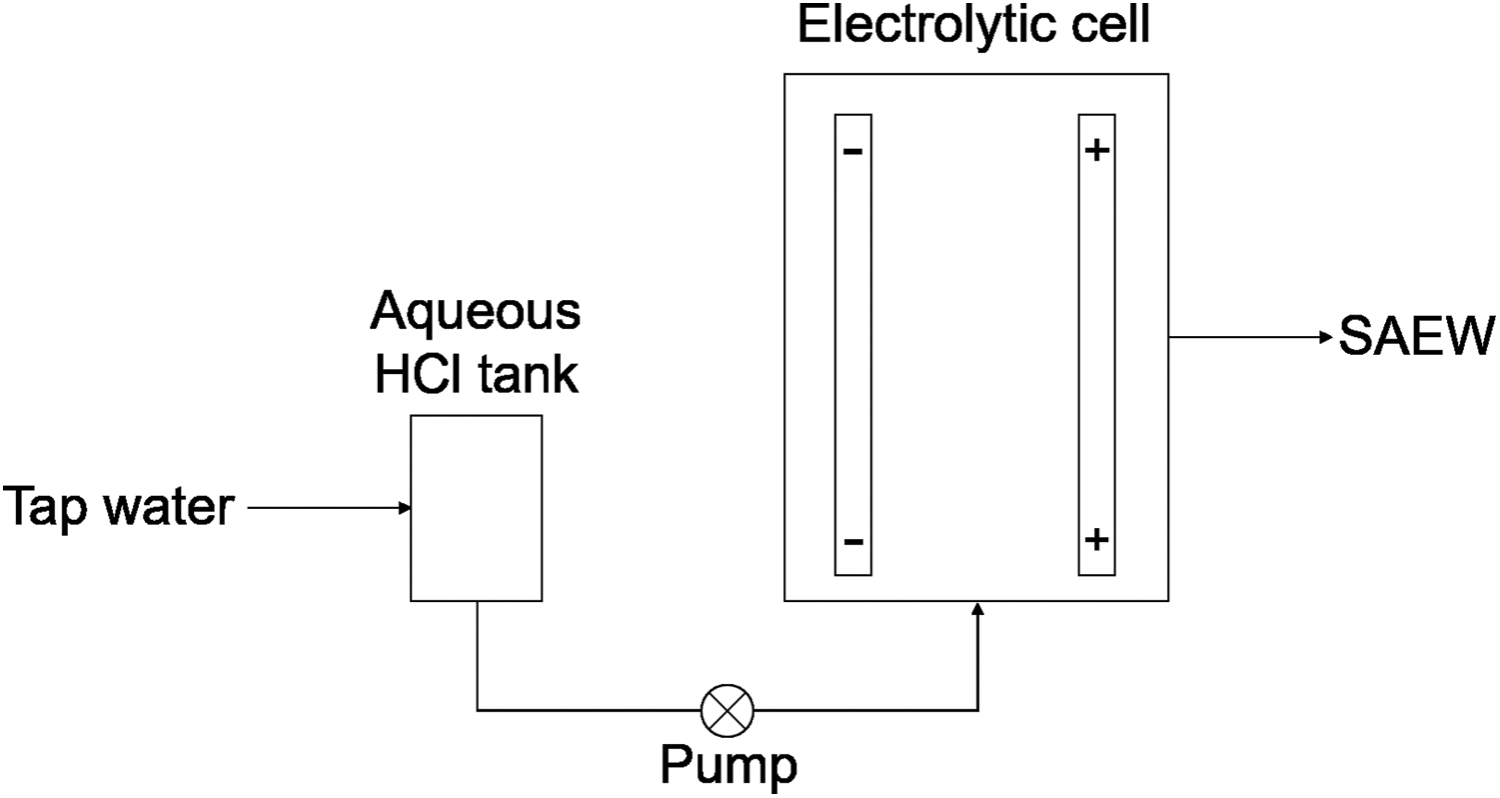
Schematic representation of SAEW production.

SAEW is produced by electrolyzing an aqueous solution of NaCl or HCl using a non-membrane electrolytic cell. In this research, we use a system which electrolyzes dilute hydrochloric acid (HCl) solution (3%) in a non-diaphragm electrolytic cell. This is because a HCl based system does not release NaCl, which is responsible for negative effects on infrastructure, such as metal corrosion. A schematic representation of the system is presented in Fig. 1. The aqueous HCl solution is supplied to the electrolytic cell where the following electrolysis reactions take place

$$2\ {\rm Cl}^{-} \rightarrow {\rm Cl}_2 + 2{\rm e}^{-}$$

On the anode, the chlorine ion is electrolyzed to chlorine and it undergoes the following reaction with water. As a result, hypochlorous acid is generated, which is the bactericidal chemical

$${\rm H}_2{\rm O} + {\rm Cl}_2\rightarrow {\rm HOCl} + {\rm H}^+ + {\rm Cl}^{-}$$

On the cathode, hydrogen gas is generated.

$$2{\rm H}^{+} + 2{\rm e}^{-}\rightarrow {\rm H}_2$$

Previous studies have shown that SAEW is suitable for the prevention and control of microorganisms due to its bactericidal activity (Cao et al., 2009; Hao et al., 2011, 2014; Issa-Zacharia et al., 2011, 2010a; Kim et al., 2019; Zhang et al., 2011; Zheng et al., 2014). SAEW poses a nearly neutral pH value, between pH 5.0 and 6.5, with available chlorine concentration usually around 30 mg/L. At this pH range, the effective form of chlorine compound is mainly hypochlorous acid (HOCl, 95%), a weak acid (p*K*_a_ = 7.53, 25 °C), which gives the antimicrobial activity (Yoshifumi, 2003). The pH of the solution has important effects on the form of chlorine compounds (ClO^−^, Cl_2_ or HOCl). At pH values above 7.5, the hypochlorous acid exists as ClO^−^ ions concentration, resulting in much lower antimicrobial activity compared to the molecule form (HOCl) (Dukan, Belkin & Touati, 1999; El-Kest & Marth, 1988a, 1988b; Huang et al., 2008). The advantages of SAEW are its qualities of being non-corrosive, environmentally friendly, stable in storage, inexpensive, ease of water reversion after use, having fewer potential health hazards due to its pH range and that it is highly effective against bacteria. Some disadvantages of the SAEW lay on the fact that users need to purchase and install an equipment to produce SAEW, with the price dependent on the required production capacity. However, there are other available options such as purchasing SAEW packs or portable machines.

There is still a lack of information and understanding on the efficiency of SAEW to inactivate microorganisms. Specifically, there is a lack of research comparing SAEW to other disinfectants such as NaOCl, the most widely used disinfectant in the food industry, at the same free chlorine concentration. Therefore, there is a need to compare the bactericidal activity of SAEW with chlorine-based disinfectants at the same free chlorine concentration. Studies conducted previously compared the antibacterial effectiveness of SAEW at 20–30 mg/L available chlorine concentration with NaOCl at 100–150 mg/L available chlorine concentration. This is because NaOCl is commonly used as a sanitizer in food processing at 50–200 mg/L of available chlorine concentration (Hao et al., 2011; Issa-Zacharia et al., 2011, 2010a, 2010b). Also, there is little, if any, research on the effective dose of SAEW in terms of concentration and duration when sprayed into the air to reduce or eliminate airborne bacteria. Thus, the objectives of this study were: (1) to quantify the bactericidal efficiency of SAEW for inactivation of *Escherichia coli* and *Pseudomonas aeruginosa*, pathogens of major public health concern because they pose high health risk and can also cause death (2) to compare the efficiency of SAEW and sodium hypochlorite (NaOCl) in reducing *E. coli* and *P. aeruginosa* at the same free chlorine concentration; (3) to determine the dose and efficiency of SAEW spray to reduce airborne bacteria by testing it against bioaerosol of *Staphylococcus epidermidis*, which is, usually, a nonpathogenic bacteria, but represents an infection risk for patients with weakened immune systems.

## Materials and Methods

### SAEW preparation

SAEW was generated by electrolysis of a mixture of aqueous dilute solution of HCl (3%) and tap water using an Apia 60 generator (Hokuty Co. Ltd., Kanagawa, Japan). The physicochemical properties of SAEW were measured immediately after production. The pH was measured using the Piccolo pH meter (Hanna Instruments Inc., Woonsocket, RI, USA). The free chlorine concentration was determined using the HI96701 Free Chlorine Photometer. The measurement range for the photometer is 0.00 to 5.00 mg/L and the resolution is 0.01 mg/L from 0.00 to 3.50 mg/L and 0.10 mg/L above 3.50 mg/L. The SAEW was used within 1 h after preparation. SAEW solutions with 0.5 mg/L (in vitro experiments) and 10 mg/L and 20 mg/L (spray experiments) were prepared by diluting SAEW in autoclaved distilled water. The free chlorine (HOCl/OCl^−^) concentration was verified three times before every experiment.

### NaOCl preparation

An analytical grade NaClO solution (Wako Pure Chemical Industries, Ltd., Osaka, Japan) containing 2.9 g/L of available chlorine was diluted in autoclaved distilled water to obtain 0.5 mg/L of available chlorine for the experiments. The available chlorine was determined by the HI96701 Free Chlorine Photometer.

### Culture preparation

Liquid-dried cultures of *E. coli* (NBRC 3972), *P. aeruginosa* (NBRC 13275) and *S. epidermidis* (NBRC 100911) were obtained from the Biological Resource Center (NBRC) of the National Institute of Technology and Evaluation (NITE), Japan. Microorganisms were revived according to L-dried culture reactivation procedures provided by the manufacturer: the L-dried culture ampoule was carefully and aseptically snap opened, a few drops of 0.9% NaCl solution was added using a sterile pasteur pipette, and then gently agitated for 2 min approximately. The suspension was placed in agar plates and left undisturbed at 35 °C for 24 h (bacterial stock). The culture medium placed in petri dishes consists of a mixture of 1 g of magnesium sulfate heptahydrate (MgSO_4_ · 7H_2_O, Nacalai Tesque, Inc., Kyoto, Japan), 10 g of hypolypepton (Nihon Pharmaceutical Co., Ltd., Tokyo, Japan), 2 g of yeast extract B2 (Oriental Yeast, Co., Ltd., Tokyo, Japan) and 15 g of agar powder (Wako Pure Chemical Industries Ltd., Osaka, Japan), in one L of distilled water. Bacterial culture of *E. coli, P. aeruginosa* and *S. epidermidis* were prepared to conduct experiments by transferring several colonies from the bacterial stock to five mL of 0.9% NaCl solution using a sterile inoculation loop and shaken using a vortex mixer (Vortex-Genie 2; Scientific Industries Inc., Bohemia, NY, USA).

**Figure 2:**
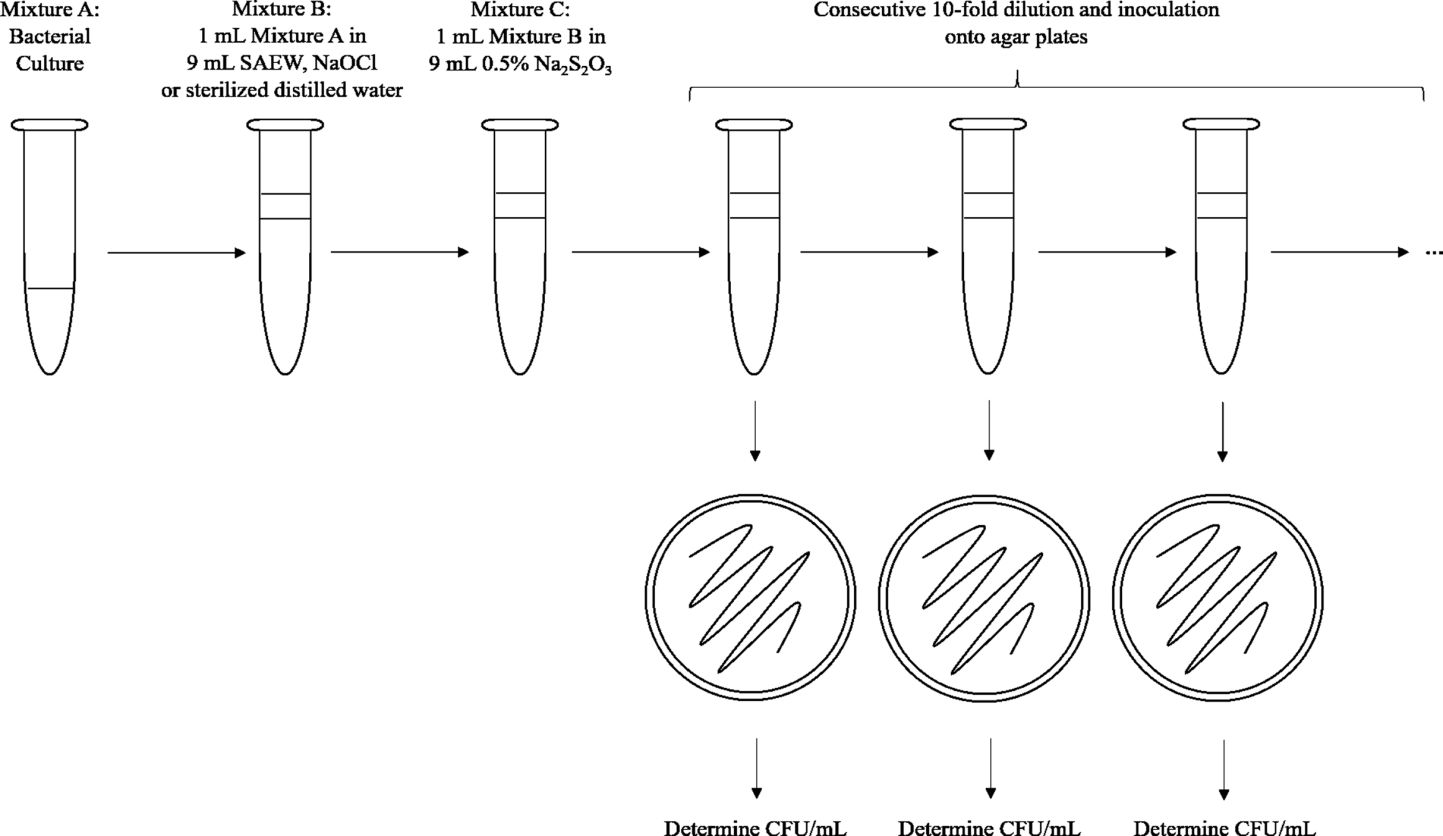
Experimental sequence for in vitro tests.

One milliliter of each bacteria suspension (approximately 8.0 log_10_ CFU/mL) was added to nine mL of sterilized water, SAEW 0.5 mg/L, or NaOCl 0.5 mg/L. The mixture was shaken using a vortex mixer and left undisturbed for 1 min. Following treatment with SAEW, inactivation experiments were conducted by transferring one mL of each treated sample to a sterile tube containing nine mL of neutralizing buffer solution (0.5% sodium thiosulfate solution, Na_2_S_2_O_3_). The tube was shaken using the vortex mixer. After 5 min of neutralization, the viable count of bacteria cells in each sample was determined by plating 0.1 mL of bacterial mixture either directly or after serial dilution (1:10) in sterile 0.9% NaCl solution. The plates were incubated at 35 °C for 24 h. After this period, the bacterial colony was counted and the bactericidal activity of SAEW and NaOCl evaluated. Microbial counts were expressed as log_10_ CFU/mL sample, with the reduction in bacterial population also calculated and presented as log_10_ CFU/mL to compare the inactivation effects of both SAEW and NaOCl. The effect of the neutralizing solution (0.5% sodium thiosulfate solution) alone was also tested on bacterial cultures to ensure that the observed bacterial reductions were solely attributed to treatment solutions and not due to sodium thiosulfate solution. Sterilized distilled water was used as control for this experiment. Each experimental case was replicated 6 times to assure repeatability. A schematic representation of the in vitro experiment is presented in Fig. 2.

### Spray experiment

**Figure 3:**
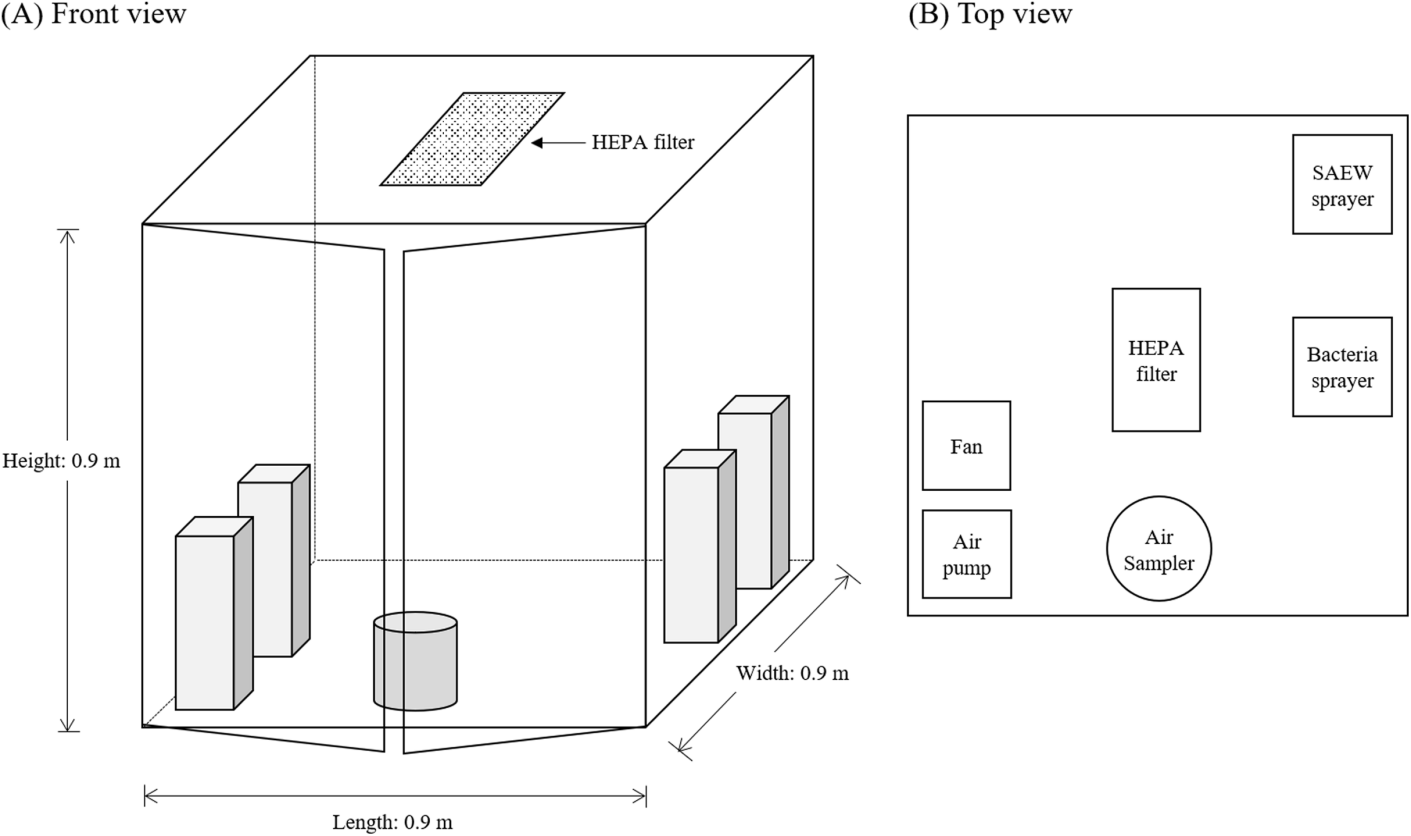
Design of spray experiments: (A) front view, (B) top view.

Spraying SAEW is potentially an effective way to decontaminate pathogenic microorganisms in the air. Experiments were conducted in a clean booth (transparent plastic clean room) 0.9 m × 0.9 m × 0.9 m in dimension, average volume of 0.729 m^3^, equipped with a HEPA filter on the top to allow only clean air enter to the room as shown in Fig. 3. An air sampler (MBS-1000 manufactured by Midori Anzen Co. Ltd., Shibuya-Ku, Japan) and a 90 mm diameter plastic plate agar culture medium were placed inside the cabin and set to collect 20 L of air after 20 min. A small fan (Eluteng, 120 mm) was placed and set at 1,500 rpm to assure uniform distribution of bacteria and SAEW during experiments. An electric air pump (SABLE, 200 L/min) was also installed inside the clean booth. *S. epidermidis* suspension (200 mL, 10^5^ CFU/mL) was prepared in a 0.9% NaCl solution and placed in a sprayer (ultrasonic humidifier, KX 80UP-VT, 90 mL/h). SAEW solution of 10 and 20 mg/L was prepared and poured in a sprayer (ultrasonic humidifier, Ottostyle Uruoi+, 300 mL/h).

After setting the system inside the clean booth and making sure that each unit could be turned on and off from outside the booth, except the air pump that was set to collect air samples after 20 min, the door was closed. The electric air pump was turned on to pump out the air inside the booth and allow clean air to enter the clean room for about 7 min. Once the air inside was clean, bacteria suspension was sprayed for 3 min at a rate of 90 mL/h (4.5 mL of bacterial suspension release). SAEW solution was sprayed following bacterial spraying in a rate of 300 mL/h for 10, 20, 30 and 60 s and was left undisturbed for 5 min before collecting air samples with the air sampler. Experiments were conducted at 25 °C and three replicate trials were done for each case.

## Results and Discussion

### In vitro results

The pH of stock solutions and the available chlorine concentration of sterilized distilled water, SAEW dilutions and NaOCl dilutions used for all experiments are presented in Table 1. SAEW had a pH value ranging between 5.5 and 5.8 and a free chlorine concentration of around 29 mg/L whereas NaOCl had a pH around 8.7 and an available chlorine concentration of 2,900 mg/L. Concentrated solutions of SAEW and NaOCl were used to prepare diluted solutions for experiments.

**Table 1:** Physicochemical properties (pH and free chlorine concentration) of tested solutions.

	Unit	Sterilized distilled water	SAEW (stock)	NaOCl (stock)
pH of stock solution	–	6.9 ± 0.5	5.6 ± 0.1	8.7 ± 0.04
Free chlorine concentration of concentrated solutions	mg/L	NA	28.9 ± 1.1	2,900
Free chlorine concentration of diluted solutions	mg/L	NA	In vitro	In vitro
			0.5	0.5
			Spray	
			10.0 ± 0.5	
			20.2 ± 0.3	

**Note:**

NA: not applicable.

Results of bacteria inactivation using 0.5 mg/L of SAEW and 0.5 mg/L of NaOCl are presented in Table 2 for *E. coli* and *P. aeruginosa*. Results obtained in this research showed that bacterial cultures were completely inactivated when using 0.5 mg/L of SAEW and partly reduced when applying 0.5 mg/L of NaOCl. SAEW with an available chlorine concentration of 0.5 mg/L with 1 min of treatment time showed a bacterial reduction capacity of more than 4.5 log_10_ CFU/mL for *E. coli* and a bacterial reduction capacity of more than 4.4 log_10_ CFU/mL for *P. aeruginosa*. On the other hand, NaOCl with an available chlorine concentration of 0.5 mg/L and also treated for 1 min showed a bacterial reduction capacity of 1.3 log_10_ CFU/mL for *E. coli* and a bacterial reduction capacity of 2.3 log_10_ CFU/mL for *P. aeruginosa*. The available chlorine in SAEW, even at low dosage, is the most important component in reducing bacteria count. Bacterial populations of 8.4 log_10_ CFU/mL for *E. coli*, and 8.5 log_10_ CFU/mL for *P. aeruginosa* were found in control experiments using sterilized distilled water. Results of in vitro experiments showed that SAEW is effective at low dosage which makes it suitable to use not only at industrial levels or factories, but also at home and public places such as restaurants where hygiene must be assured. SAEW can also be considered as an alternative solution to conventional sanitizers such as NaOCl which is usually applied in higher dosage (Issa-Zacharia et al., 2010b; Koseki et al., 2004).

**Table 2:** Results of bacterial reduction or inactivation using SAEW and NaOCl. The experiments were repeated 5–6 times.

	Sterilized distilled water	SAEW 0.5 mg/L	NaOCl 0.5 mg/L
*Escherichia coli*	0.06 ± 0.03	>4.49 ± 0.003	1.28 ± 0.91
*Pseudomonas aeruginosa*	0.15 ± 0.11	>4.38 ± 0.005	2.31 ± 0.67

**Note:**

Unit: log_10_ CFU/mL.

The difference in bactericidal activity between SAEW and NaOCl lays on the presence of hypochlorous acid (HOCl), a very effective disinfectant, rather than the total available chlorine concentration. This is true even at concentrations of less than 0.1 mg/L (Ludovici, Phillips & Jeter, 1975). SAEW consists mainly of HOCl, whereas the ion OCl^−^ is the major chlorine specie present at the pH in commercial NaOCl dilutions (50–200 mg/L of available chlorine concentrations). It is believed that HOCl’s bactericidal activity is due to its ability to diffuse through the microbial cell, causing damage to the plasma membrane and DNA, and also inhibiting enzyme activity essential for growth (Fukuzaki, 2006). Conversely, the poor germicidal activity of the OCl^−^ specie is because penetration of this ion is prevented by the hydrophobic layer of microbe’s plasma membrane (Fukuzaki, 2006).

Nan et al. (2010), Kim et al. (2019) and Kiura et al. (2002) analyzed the bacterial cellular morphology before and after treatment with solutions containing free chlorine by transmission electron microscopy. Nan et al. (2010) and Kim et al. (2019) found that *E. coli* cells were remarkably damaged, destroyed or deformed after exposure to SAEW with free chlorine concentrations between 10 and 60 mg/L. Kiura et al. (2002) observed damage of the outer membrane of *P. aeruginosa* and inactivation of cytoplasmic enzyme after exposure to free chlorine concentration solutions, which, according to this research team, are clear indicators of the bactericidal activity of free chlorine solutions. The bactericidal effect of SAEW lies in the synergy between high oxidation-reduction potential (ORP, equal to or greater than 900 mV) and HOCl. First, a series of ORP reactions take place which damage the outer and inner membrane and inactivate the defense mechanism of bacteria. Subsequently, HOCl can penetrate the bacterial cell and oxidize it, resulting in cell inactivation, inhibition of ATP generation or death (Issa-Zacharia et al., 2011).

### Spray experiments

**Figure 4:**
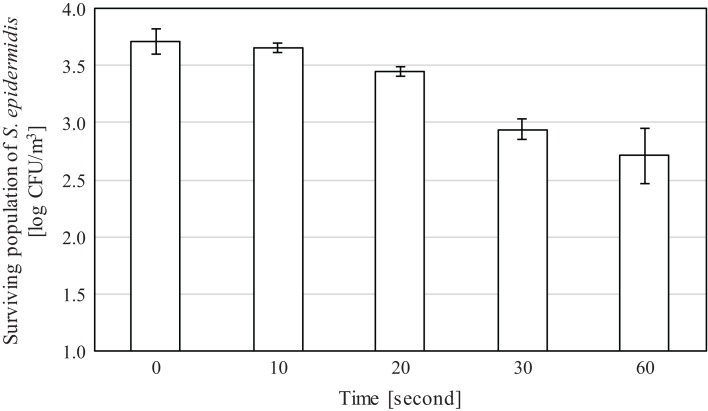
Bacteria population after spraying 10 mg/L of SAEW. The bacteria population decreases after spraying 10 mg/L of SAEW for 0–60 s.

**Figure 5:**
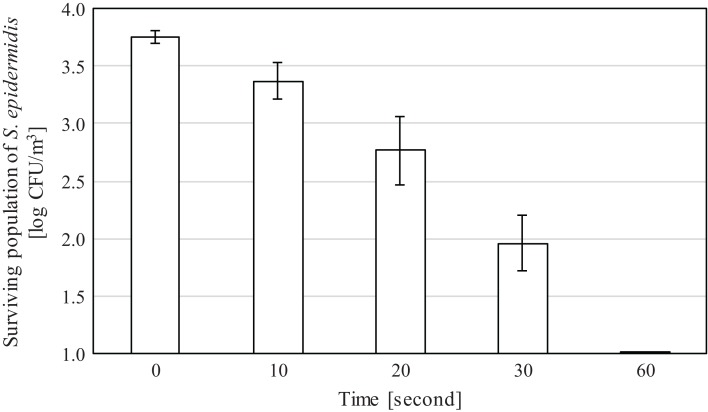
Bacteria population after spraying 20 mg/L of SAEW. The bacteria population decreases after spraying 20 mg/L of SAEW for 0–60 s.

In the spray experiments, a great reduction in the population of the bacteria *S. epidermidis* was observed after spraying 10 and 20 mg/L of SAEW. This indicates that SAEW can be probably used as an alternative to purifying the air in food processing factories, agricultural facilities, hospitals, care homes, fruit and vegetable markets, restaurants, etc. even at lower concentrations and for short periods of time. Results presented in Figs. 4 and 5 showed a gradual decrease in bacterial population as the SAEW spraying time was increased from 10 to 60 s. The average initial bacterial concentration for experiments using 10 mg/L was 3.72 log_10_ CFU/m^3^, whereas for experiments using 20 mg/L, it was 3.76 log_10_ CFU/m^3^. Reduction in terms of log_10_ CFU/m^3^ and percentages are presented in Table 3. From this table, a decrease of 0.06, 0.26, 0.77 and 1.00 log_10_ CFU/m^3^ or 12%, 44%, 82% and 89% was observed in bacteria population after spraying 10 mg/L of SAEW for 10, 20, 30 and 60 s respectively at a 300 mL/h rate. Also, a decrease in 0.39, 0.99, 1.80 and 3.76 log_10_ CFU/m^3^ or 56%, 88%, 98% and 100% was observed in bacteria population after spraying 20 mg/L of SAEW for 10, 20, 30 and 60 s respectively at a 300 mL/h rate. This indicates that SAEW can be sprayed at lower concentrations and that continuous supply is probably not required. From the overall results, it appears that 20 mg/L of SAEW for 60 s is enough to almost completely eliminate the bacteria in the air.

**Table 3:** Results of spray experiments in both log scale and percentage. The experiments were repeated 3 times.

Time	SAEW 10 mg/L		SAEW 20 mg/L	
	Reduction log_10_ CFU/m^3^	Reduction percentage	Reduction log_10_ CFU/m^3^	Reduction percentage
10 s	0.06 ± 0.06	11.6 ± 13.4	0.39 ± 0.21	55.7 ± 19.9
20 s	0.26 ± 0.12	43.6 ± 16.3	0.99 ± 0.28	88.3 ± 7.8
30 s	0.77 ± 0.20	81.8 ± 9.0	1.80 ± 0.25	98.3 ± 0.8
60 s	1.00 ± 0.27	88.9 ± 5.5	3.76 ± 0.06	100 ± 0.0

A few studies reported on the application of SAEW spray for the reduction of airborne bacteria in animal housing. Weichao et al. (2016) reported on the optimization of different operating parameters (nozzle orifice diameter and spray pressure) when using SAEW spray with 30 mg/L of available chlorine concentration to improve the indoor air environment in animal housing. Hao et al. (2014) studied the distribution and subsequent reduction of airborne bacteria and fungi after 30 min of SAEW spraying in tunnel ventilated layer breeding houses. They found that both bacteria and fungi population sharply decreased after 30 min of SAEW with 250 mg/L of available chlorine concentration exposure. Zheng et al. (2013) reported on the use of SAEW spray with 150–250 mg/L of available chlorine concentration in laying-hen houses for 30 min and found that the efficiency of SAEW on the inactivation of pure cultures increased with increasing available chlorine concentration. These research teams held experiments using SAEW with available chlorine concentrations from 30 to 250 mg/L and for long periods of time. Therefore, our contribution lay on the fact that SAEW spray with much lower concentration (10 and 20 mg/L of available chlorine concentration) and reduced exposure time (intermittent or not continuous application) is effective and can be applied to public places where clean air is necessary, while guaranteeing people’s health and safety needs.

## Conclusions

The results obtained in this study demonstrated that SAEW exhibits higher bactericidal activity compared to NaClO when tested at 0.5 mg/L and was effective in reducing and/or eliminating *E. coli* and *P. aeruginosa*.

*Escherichia coli* counts decreased more than 4.5 log_10_ CFU/mL when SAEW was used and 1.3 log_10_ CFU/mL when using NaOCl, both with the same available chlorine of 0.5 mg/L. *P. aeruginosa* counts decreased more than 4.4 log_10_ CFU/mL when SAEW was used and 2.3 log_10_ CFU/mL when using NaOCl, both with the same available chlorine of 0.5 mg/L. This indicates that at the same chlorine concentration, SAEW is more effective in reducing or eliminating bacterial count.

Spray experiments showed that bacterial counts gradually decreased as SAEW spray was applied for 10, 20, 30 and 60 s. After spraying 10 mg/L of SAEW for 60 s, *S. epidermidis* counts decreased from 3.7 log_10_ CFU/m^3^ to 2.8 log_10_ CFU/m^3^. After spraying 20 mg/L of SAEW for 60 s, bacterial count decreased from 3.8 log_10_ CFU/m^3^ to 0 CFU/m^3^. The latter result indicates that 1 min of 20 mg/L of SAEW is enough to eliminate 3.8 log_10_ CFU/m^3^ of *S. epidermidis*.

Considering that SAEW is environmentally friendly and poses low potential for damage to human health, it could be used as an effective disinfectant, in both solution and spray form, in the food industry, industry in general, or at home.

## Supplemental Information

10.7717/peerj.8593/supp-1 Supplemental Information 1 Raw Data 1: Physicochemical Parameters. pH values and Cl concentrations.

10.7717/peerj.8593/supp-2 Supplemental Information 2 Raw Data 2: In vitro experiment results. Comparison of the bactericidal effect of SAEW and sodium hypochlorite against *Escherichia coli* and *Pseudomonas aeruginosa*.

10.7717/peerj.8593/supp-3 Supplemental Information 3 Raw Data 3: Spray experiment results. Evaluation of the bactericidal effect of SAEW (10 ppm and 20 ppm) against *Staphylococcus epidermidis*.

## Additional Information and Declarations

### Data Availability

The following information was supplied regarding data availability: The raw measurements are available in the Supplemental Files.
